# Job-exposure matrix for historical exposures to rubber dust, rubber fumes and n-Nitrosamines in the British rubber industry

**DOI:** 10.1136/oemed-2018-105182

**Published:** 2019-02-16

**Authors:** Mira Hidajat, Damien Martin McElvenny, William Mueller, Peter Ritchie, John W Cherrie, Andrew Darnton, Raymond M Agius, Hans Kromhout, Frank de Vocht

**Affiliations:** 1 Population Health Sciences, Bristol Medical School, University of Bristol, Bristol, UK; 2 Research Division, Institute of Occupational Medicine, Edinburgh, UK; 3 Institute of Biological Chemistry, Biophysics and –Bioengineering, Heriot Watt University, Edinburgh, UK; 4 Statistics and Epidemiology Unit, Health and Safety Executive, Bootle, UK; 5 Centre for Occupational and Environmental Health, Centre for Epidemiology, School of Health Sciences, University of Manchester, Manchester, UK; 6 Environmental Epidemiology Division, Institute for Risk Assessment Sciences, Utrecht University, Utrecht, The Netherlands

**Keywords:** job-exposure matrix, JEM, rubber industry, industrial cohort study, rubber fumes, rubber dust, nitrosamines, occupational exposures

## Abstract

**Objectives:**

To develop a quantitative historical job-exposure matrix (JEM) for rubber dust, rubber fumes and n-Nitrosamines in the British rubber industry for 1915–2002 to estimate lifetime cumulative exposure (LCE) for a cohort of workers with 49 years follow-up.

**Methods:**

Data from the EU-EXASRUB database—rubber dust (n=4157), rubber fumes (n=3803) and n-Nitrosamines (n=10 115) collected between 1977 and 2002—were modelled using linear mixed-effects models. Sample year, stationary/personal measurement, industry sector and measurement source were included as fixed explanatory variables and factory as random intercept. Model estimates and extrapolations were used to construct a JEM covering all departments in both sectors of the rubber manufacturing industries for the years 1915–2002. JEM-estimates were linked to all cohort members to calculate LCE. Sensitivity analyses related to assumptions about extrapolation of time trends were also conducted.

**Results:**

Changes in rubber dust exposures ranged from −6.3 %/year (crude materials/mixing) to −1.0 %/year (curing) and −6.5 %/year (crude materials/mixing) to +0.5 %/year (finishing, assembly and miscellaneous) for rubber fumes. Declines in n-Nitrosamines ranged from −17.9 %/year (curing) to −1.3 %/year (crude materials and mixing). Mean LCEs were 61 mg/m^3^-years (rubber dust), 15.6 mg/ m^3^-years (rubber fumes), 2483.2 µg/m^3^-years (n-Nitrosamines sum score), 18.6 µg/m^3^-years (*N*-nitrosodimethylamine) and 15.0 µg/m^3^-years (*N-*itrosomorpholine).

**Conclusions:**

All exposures declined over time. Greatest declines in rubber dust and fumes were found in crude materials and mixing and for n-Nitrosamines in curing/vulcanising and preprocessing. This JEM and estimated LCEs will allow for evaluation of exposure-specific excess cancer risks in the British rubber industry.

**Key messages**

**What is already known about this subject?**

- This paper is the first to develop a quantitative historical job-exposure matrix (JEM) describing historical (1915–2002) occupational exposures (rubber dust, rubber fumes and n-Nitrosamines) exposures for both the British tyre production and general rubber goods manufacturing.

**What are the new findings?**

- All exposures declined over time. Greatest declines in rubber dust and fumes were found in crude materials and mixing and for n-Nitrosamines in curing/vulcanising and preprocessing.
- The estimated lifetime cumulative exposure (LCE) based on the JEM for this cohort appeared to be relatively insensitive to choices on extrapolation of time time-trends beyond the range of the measurement data.
- The JEM enabled estimation of exposure-specific LCE estimation for a cohort of British rubber manufacturing workers with 49 years of follow-up for mortality.

**How might this impact on policy or clinical practice in the foreseeable future?**

- This JEM and estimated LCEs allow for evaluation of exposure-specific excess cancer risks in the British rubber industry.

## Introduction

Employment in the rubber manufacturing industry has been shown to cause cancer as a result of exposures generated during the rubber production process, and which include n-Nitrosamines, rubber (process) dust, rubber fumes, n-Nitrosamines, polyaromatic hydrocarbons including β-naphthylamine, phthalates, aromatic amines and solvents including benzene. The International Agency for Research on Cancer’s (IARC) Monograph Working Group concluded there is sufficient evidence in humans for the carcinogenicity of occupational exposures in the rubber-manufacturing industry (Group 1), with causal relationships established for cancers of the urinary bladder, lung and stomach and leukaemia, multiple myeloma and non-Hodgkin’s lymphoma as well as suggestions of increased risks for cancers of the prostate, oesophagus and larynx.^1^ The Working Group also specifically classified carcinogens 2-Naphthylamine (bladder cancer), which was a contaminant in an antioxidant, and benzene (leukaemia), which was used as a solvent for rubber metal bonds, as Group 1 carcinogens, although the former was removed from the UK production process in 1949 and indicated that it remained difficult to isolate other specific exposure-carcinogenic effect associations with a sufficient degree of confidence.^1^

Risks differ between countries, factories, departments, workers and over time, which is probably indicative of specific occupational exposures being the causal agents for specific cancers. Generally, exposures to rubber dust, which consists of particles arising from raw and synthetic materials as well as additives and fillers used to create the final rubber product,^1 2^ is highest in the beginning stage of the manufacturing process where raw materials are transported, weighed, mixed with other chemicals and stored.^3^ Rubber fumes are generated when rubber mixtures are heated and cured along with other chemical accelerators and vulcanisation agents.^1 4^ *N*-Nitrosamines are produced during the vulcanising stage of the manufacturing process where rubber mixtures are heated along with other chemical accelerators and vulcanisation agents, such as tetramethylthiuram disulfide, zinc-diethyldithiocarbamate and morpholino mercapto-benzothiazole^5^ which due to nitrosation produce *n*-Nitrosamines. The most-encountered nitrosamine in rubber manufacturing factories is *n*-nitrosodimethylamine (NDMA), but other nitrosamines identified include *n*-nitrosodiethylamine (NDEA), *n*-nitrosodibutylamine, *n*-nitrosopiperidine (NPIP) and *n*-nitrosomorpholine (NMor).^1^

To evaluate the association between lifetime cumulative exposures (LCEs) to specific occupational exposures encountered in the rubber industry and cancer mortality, we have updated a cohort study of UK rubber manufacturing workers established in 1967^6^ up to 2015 (l 49 years mortality follow-up). External Standardised Mortality Ratio analyses of this updated cohort are presented in a companion paper.^7^ In this paper, we present the methodology for the development of a historical, quantitative, job-exposure matrix (JEM) covering the cohort members' employment period (1915–2002), and which serves as the basis for a quantitative evaluation of cancer mortality risk and assessment of exposure-response associations to the specific carcinogens rubber dust, rubber fumes and n-Nitrosamines. This study represents the first historical quantitative JEM approach with estimations of LCE for a UK rubber manufacturing worker cohort.

## Materials and methods

### Data

Measurements of personal exposure and area air concentrations of rubber dust, rubber fumes and n-Nitrosamines have been collated in the EXASRUB database.^8^ In short, the EXASRUB database is a compilation of air concentration measurements in rubber factories in Europe and includes measurements that were collected for several purposes, including research and compliance testing. For analyses of rubber dust (n=4157) and rubber fumes (n=3803), we used data from the UK, which were collected by the British Rubber Manufacturing Association (BRMA) and present within the HSE’s National Exposure Database (HSE-NEDB) in the period of 1977–2002 and which largely overlap with data used in a previous study.^3^ For analyses of n-Nitrosamines, due to the small sample size, we supplement measurements from the UK (n=84) with data from Germany (n=1939), where most n-Nitrosamines measurements in the EU-EXASRUB were collected. Industries in both countries were broadly comparable, while previous exploration of the data indicated that point estimates were generally similar and confidence intervals of estimates for average exposure overlapped.^9^

Measurements of rubber dust, rubber fumes, the sum of the five most prevalent n-Nitrosamines (sum5-Nitrosamines; that is, NDMA, NMor, NPIP, NDEA and NDBA) and individually for the two most prevalent specific nitrosamines, NDMA and NMor were analysed. Q-Q plots were explored and values >99th percentile of each exposure were removed prior to analyses because we could not evaluate whether these outliers were genuine (although unlikely high) measurements or erroneous. For rubber dust and rubber fumes, measurements below the 0.1 mg/m^3^ limit of detection (LOD) were imputed with a single value^10^ between 0.01 and 0.1 mg/m^3^, similar to previous work,^4^ assuming a uniform distribution. Similar to previous work,^9^ NDMA and NMor values below the LOD of 0.3 µg/m^3^ were imputed with a single value between 0.01 and 0.3 µg/m^3^. The quality of these data was evaluated using Benford’s law and indicated any observed deviations could be attributed to imputation and replacement methods.^11^

In the EXASRUB dataset, jobs and tasks were classified based on the hierarchical BRMA classification.^4^ To enable estimation of longitudinal JEMs, comparable departments were aggregated into four groups (frequencies in online supplementary table S5): ‘Raw Materials and Mixing’ is composed of BRMA job codes 1 (crude materials and storage) and 2 (compounding and mixing); ‘Pre-processing and Assembly’ comprises BRMA job codes 3 (solutions, cements, latex and foam processing), 4 (extrusion, calendaring and stock preparation) and 5 (component building and assembly); ‘Curing/Vulcanising’ is composed of BRMA job code 6 (curing or vulcanizing) and ‘Finishing, Assembly and Miscellaneous’ is composed of BRMA job codes 7 (inspection, painting, finishing repairs), 8 (storage of finished goods, packing, dispatch), 9 (site workers, drivers, cleaners, laboratory control), 10 (engineering services, building maintenance) and 11 (non-process workers in the factory environment).

10.1136/oemed-2018-105182.supp1 Supplementary data

Other covariates in the statistical modelling were year of sampling, industry sector (tyre production or general rubber goods (GRG) manufacturing) and stationary or personal sampling. For rubber dust and fumes, an indicator variable for the measurement source, that is, BRMA or HSE-NEDB, based on previous evidence,^3^ and for n-Nitrosamines, a variable to indicate whether measurements were from the UK or Germany were included. Other exposure determinants (like measurement device and strategy) or individual behavioural or lifestyle factors such as PPE were unavailable in the EXASRUB database.

### Statistical analysis; construction of the JEM

We modelled the log-transformed concentration of each agent using a linear mixed-effect model with sample year and industry sector and a random intercept by factory (Eq. (1)).

(1) $$\begin{array}{ll} y_{ij}= & \beta_{0}+\beta_{1}SAMPLEYR+\beta_{2}SECTOR+\\ \; & \beta_{3}PERSONAL+\beta_{4}SOURCE+u_{j}+\varepsilon_{ij} \end{array}$$

where *y_ij_*=loge concentration in mg/m^3^ for rubber dust and fumes, NDMA, NMor and nitrosamines sum score for *i*=1, 2,…, *n_i_* factories and *j*=1, 2,…, *n_j_* measurements nested within factories; *β_0_*=intercept; *β_1_SAMPLEYR_ij_*=fixed effects of the time trend; *β_2_SECTOR_ij_=*fixed effects of the industry sector; *β_3_PERSONALij*=personal or stationary measurement; *β_4_SOURCE_ij_*=fixed effect for source of measurement (BRMA/HSE for rubber dust or fumes and UK/Germany for n-Nitrosamines); *u_j_=*random intercept adjustment for measurements at the same factory and *ε_ij_* residual variance.

We ran separate models for each department group. Using results from each model, geometric means (GM) of each exposure were calculated as estimates of average personal exposure a worker would have received for each year, industry sector and department combination. Models were projected backward from 1977 or 1981 to 1915 and forward from 2001 or 2002 to 2015 assuming that average trend from the first or last available measurements continuous to enable the calculation of lifetime exposure to each agent for the cohort. However, because extrapolation of lognormal exposures as far back as 1915 results in impossibly high estimates of average exposure, rubber dust and rubber fumes were capped at 50 mg/m^3^, similar to the methodology used in the development of another quantitative job-exposure matrix SYN-JEM,^12^ while n-Nitrosamines, for lack of previous reference methods, were capped at twice the highest estimate of average exposures for a year with measurements. To evaluate these backcasting assumptions, we conducted sensitivity analysis in which a constant average exposure level was assumed from the first year of available measurement in the EXASRUB database. A comparable strategy was previously used to evaluate assessment of historical exposures in the asphalt paving industry.^13^

Last, these model estimates were used to derive a quantitative, historical JEM by transforming back from log-space to provide estimates of GM exposure for each agent, industry sector, department and year combination. These personal exposure estimates were subsequently linked at an individual level to the cohort of workers employed for at least 1 year in rubber factories in Great Britain on 1 February 1967 (n=36 441); more on the study cohort can be found in the companion papers.^7 14^ For each worker, LCE was calculated by summing the exposure level to each agent from the start year of employment to the retirement year (assuming each worker retires at age 70) and is expressed as mg/m^3^-years for rubber dust and fumes and μg/m^3^-years for n-Nitrosamines, respectively.

All analyses were conducted in Stata 14.2 (Stata, release 14, 2018).

## Results

Numbers of measurements per department and year for the exposures are shown in online supplementary tables S1–S3. Estimates for rubber dust were based on 4157 measurements collected in 1977–2002 (11.3% below LOD), of which 58% were personal measurements. 91% of measurements were from BRMA and 61% were from the tyres industry. The number per department group ranged from 606 to 2005 (table 1). Results of the linear mixed effects models are provided in online supplementary tables S4–S8. GM for rubber dust was 0.58 mg/m^3^. GM levels in crude materials/mixing and in finishing (0.41 mg/m^3^) were on average about 25% higher than in preprocessing/assembly and curing. Average exposure was more than 2.5 times higher in GRG compared with tyres production and was also higher in HSE-collected measurements than in those collected by the rubber manufacturing industry (2.71 mg/m^3^ vs 1.76 mg/m^3^, respectively). Exposure declined by about 6% (95% CI −5% to −8%) annually in Crude Materials/Mixing and was −1% per year in the other departments (online supplementary table S9). Conversely (table 2), JEM estimates indicated average exposure levels of 50 mg/m^3^ from 1915 to 1935 (capped), followed by a decline from 37.5 mg/m^3^ in 1940 to 0.7 mg/m^3^ in 2002 and from 50 mg/m^3^ to 1.1 mg/m^3^ in Crude Materials/Mixing department, tyres and GRG production sectors, respectively. In Pre-processing/Assembly, average exposure declined from 1.0 to 0.3 mg/m^3^ in tyres and 1.5 to 0.5 mg/m^3^ in GRG; in Curing from 0.8 to 0.3 and 0.9 to 0.4 mg/m^3^, respectively and in Finishing from 1.6 to 0.6 and 1.0 to 0.3 mg/m^3^ in tyres and GRG, respectively.

**Table 1:** Distributions of rubber dust, rubber fume and n-Nitrosamines measurements

	Rubber dust (mg/m^3^)				Rubber fumes (mg/m^3^)				n-Nitrosamines sum score (μg/m^3^)				NDMA* (μg/m^3^)				NMor† (μg/m^3^)			
	N	AM	GM	GSD	N	AM	GM	GSD	N	AM	GM	GSD	N	AM	GM	GSD	N	AM	GM	GSD
Number of measurements	4157	1.84	0.58	4.6	3803	0.57	0.26	3.51	2023	7.87	0.56	1.41	2023	0.32	0.16	2.81	2025	0.27	0.16	2.70
Below LOD‡	11.3%				21.9%				NA				88.7%				85.7%			
Sector																				
General rubber goods	1607	2.68	1.07	4.01	1987	0.76	0.39	3.29	787	14.82	1.06	1.73	795	0.29	0.16	2.72	793	0.29	0.16	2.82
Tyres	2550	1.31	0.40	4.42	1816	0.35	0.17	3.23	1236	3.44	0.24	1.04	1228	0.33	0.16	2.87	1232	0.25	0.16	2.62
P value	0.053				0.002				<0.001				0.012				0.128			
Aggregated BRMA§ Departments																				
Crude materials and mixing	2005	2.62	0.41	4.93	1107	0.49	0.23	3.76	85	1.13	−0.06	0.54	86	0.20	0.15	2.86	86	0.28	0.16	2.92
Preprocessing and assembly	616	1.23	0.32	3.73	515	0.34	0.16	3.43	250	5.68	0.26	1.16	249	0.35	0.16	2.76	249	0.23	0.15	2.57
Curing, Vulcanising	930	0.74	0.33	4.07	1716	0.67	0.34	3.38	1371	9.77	0.72	1.53	1373	0.31	0.16	3.19	1379	0.26	0.16	2.65
Finishing, assembly and miscellaneous	606	1.56	0.41	4.17	465	0.6	0.26	3.28	317	3.18	0.23	0.97	315	0.33	0.16	2.35	311	0.32	0.17	2.98
P value	<0.001				0.006				0.392				0.074				0.057			
Sample type																				
Personal	2416	2.53	1.00	3.95	2492	0.69	0.35	3.28	1099	10.43	0.57	1.57	1119	0.22	0.14	2.47	1114	0.21	0.14	2.43
Stationary	1741	0.87	0.28	4.09	1311	0.33	0.15	3.29	924	4.82	0.54	1.18	904	0.43	0.18	3.17	911	0.34	0.18	2.99
P value	<0.001				<0.001				0.388				<0.001				<0.001			
Dataset																				
BRMA§	3799	1.76	0.55	4.61	2494	0.32	0.17	3.08												
HSE-NEDB¶	358	2.71	1.15	3.84	3803	1.02	0.59	2.97												
P value	0.434				0.008															
Country																				
UK**	4157	1.84	0.58	4.6	3803	0.57	0.26	3.51	84	3.67	0.34	1.01	81	0.88	0.35	3.89	83	0.35	0.17	3.01
Germany	0				0				1939	8.05	0.57	1.42	1942	0.29	0.15	2.73	1942	0.26	0.16	2.69
P value	N/A				N/A				0.590				0.010				0.628			

* *N*-nitrosodimethylamine.

†*N*-nitrosomorpholine.

‡Limit of detection.

§ British Rubber Manufacturing Association.

¶British Health and Safety Executive National Exposure Database.

**Only UK data were used for rubber fumes and rubber dust analyses.

AM, arithmetic mean; GM, geometric means; GSD, geometric standard deviation; LOD, limit of detection; NDMA, *N*-nitrosodimethylamine; NMor, *N*-nitrosomorpholine.

**Table 2:** Job exposure matrix for rubber dust and rubber fumes (mg/m^3^) in the tyre and GRG industries

Rubber dust									Rubber fumes							
Department	Crude materials and mixing		Preprocessing and assembly		Curing/Vulcanising		Finishing, assembly and miscellaneous		Crude materials and mixing		Preprocessing and assembly		Curing/Vulcanising		Finishing, assembly and miscellaneous	
Year	Tyres	GRG	Tyres	GRG	Tyres	GRG	Tyres	GRG	Tyres	GRG	Tyres	GRG	Tyres	GRG	Tyres	GRG
1915	50.00	50.00	0.96	1.49	0.82	0.95	1.62	0.95	50.00	50.00	0.93	0.80	1.48	2.06	0.12	0.13
1920	50.00	50.00	0.91	1.40	0.78	0.90	1.52	0.90	37.83	37.96	0.82	0.71	1.31	1.83	0.12	0.14
1925	50.00	50.00	0.86	1.32	0.74	0.86	1.43	0.84	27.05	27.15	0.73	0.63	1.16	1.63	0.12	0.14
1930	50.00	50.00	0.81	1.25	0.70	0.81	1.35	0.79	19.35	19.42	0.64	0.55	1.03	1.45	0.13	0.15
1935	50.00	50.00	0.76	1.18	0.66	0.77	1.27	0.75	13.84	13.88	0.57	0.49	0.92	1.29	0.13	0.15
1940	37.49	50.00	0.72	1.11	0.63	0.73	1.19	0.70	9.89	9.93	0.50	0.43	0.82	1.14	0.13	0.15
1945	27.13	43.90	0.68	1.05	0.60	0.70	1.12	0.66	7.08	7.10	0.45	0.38	0.73	1.01	0.14	0.16
1950	19.64	31.77	0.64	0.99	0.57	0.66	1.06	0.62	5.06	5.08	0.39	0.34	0.64	0.90	0.14	0.16
1955	14.22	23.00	0.60	0.93	0.54	0.63	0.99	0.59	3.62	3.63	0.35	0.30	0.57	0.80	0.14	0.16
1960	10.29	16.65	0.57	0.88	0.51	0.60	0.93	0.55	2.59	2.60	0.31	0.27	0.51	0.71	0.15	0.17
1965	7.45	12.05	0.54	0.83	0.49	0.57	0.88	0.52	1.85	1.86	0.27	0.23	0.45	0.63	0.15	0.17
1970	5.39	8.72	0.51	0.78	0.46	0.54	0.83	0.49	1.32	1.33	0.24	0.21	0.40	0.56	0.15	0.18
1975	3.90	6.31	0.48	0.74	0.44	0.51	0.78	0.46	0.95	0.95	0.21	0.18	0.36	0.50	0.16	0.18
1980	2.83	4.57	0.45	0.70	0.42	0.49	0.73	0.43	0.68	0.68	0.19	0.16	0.32	0.44	0.16	0.19
1985	2.04	3.31	0.43	0.66	0.40	0.46	0.69	0.41	0.48	0.49	0.17	0.14	0.28	0.39	0.16	0.19
1990	1.48	2.39	0.40	0.62	0.38	0.44	0.65	0.38	0.35	0.35	0.15	0.13	0.25	0.35	0.17	0.20
1995	1.07	1.73	0.38	0.59	0.36	0.42	0.61	0.36	0.25	0.25	0.13	0.11	0.22	0.31	0.17	0.20
2000	0.78	1.25	0.36	0.55	0.34	0.40	0.57	0.34	0.18	0.18	0.12	0.10	0.20	0.28	0.18	0.21
2002	0.68	1.10	0.35	0.54	0.33	0.39	0.56	0.33	0.16	0.16	0.11	0.09	0.19	0.26	0.18	0.21

GRG, general rubber good.

GM rubber fume exposure in the GRG sector was 0.26 mg/m^3^, based on 3803 measurements (66% personal) collected between 1977 and 2002 of which 21.9% were below the detection level and had to be imputed. Thirty-four per cent of measurements were from the HSE-NEDB database, with average exposure levels approximately 3.5 times higher than for those collected by the BRMA. Average exposures were 2.3 times higher in GRG compared with tyres production and ranged from 0.16 mg/m^3^ in Pre-processing/Assembly to 0.34 mg/m^3^ in Curing. Time trends across departments were more variable than for rubber dust, and were −7% (95% CI −5% to −9%) annually in Crude Materials, −3 %/year (95% CI −5% to +0.01%) in Pre-Processing/Assembly, −2 %/year (95% CI −0.4% to −0.9%) in Curing, and +0.5% (95%CI −2.2% to 3.2%) in Finishing (online supplementary table S9). Conversely (table 2), JEM estimates of average exposure declined from 50 mg/m^3^ (capped 1915–1977) in 1915 to 0.2 mg/m^3^ in 2002 in the crude materials department group in sectors. In the other department groups, average exposure declined from 0.9 to 0.1 mg/m^3^ and 0.8 to 0.1 mg/m^3^ in tyres and GRG, respectively (Pre-Processing); from 1.5 to 0.2 mg/m^3^ and 2.1 to 0.3 mg/m^3^ in Curing and was relatively stable 0.1 to 0.2 mg/m^3^ in Finishing.

A total of 2023 measurements were available that allowed for combining of individual Nitrosamines (sum score) collected between 1983 and 2001 and of which 88.7% and 85.7% were <LOD, of which 4% were from the UK (see table 1). Fifty-four per cent of these were personal measurements, with average exposure comparable to stationary measurements (0.57 and 0.54 µg/m^3^, respectively). Average exposure was about 4.5 times as high in the GRG compared with the tyres industry, and highest average exposure was found in Curing (0.72 µg/m^3^). Average trends over time (online supplementary table S9) indicated reductions of 1.3% in the crude materials departments to as much as 18% annually in curing for the sum score (table 3). No significant exposure reductions were observed for individual nitrosamines, with the exceptions of NDMA in curing (−6 %/year) and NMor in pre-processing (−4 %/year) (table 4).

**Figure 1:**
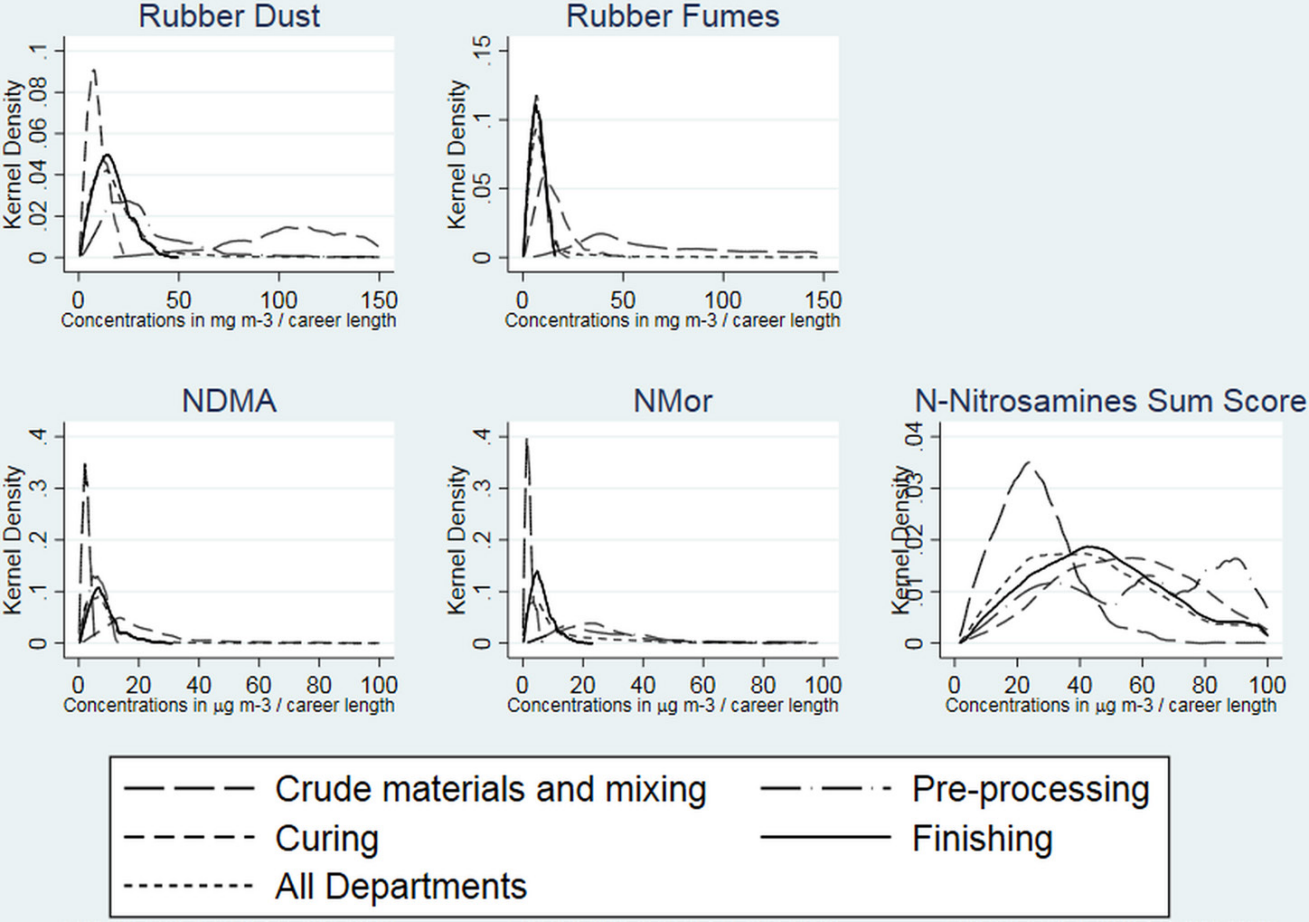
LCE to rubber dust, rubber fumes and n-Nitrosamines. LCE, lifetime cumulative exposure; NDMA, *N*-nitrosodimethylamine; NMor, *N*-nitrosomorpholine.

LCE of the cohort of 36 441 workers (59% in GRG) is shown graphically in figure 1, with summary statistics in online supplementary tables S10–14. Rubber dust LCE ranged from 1.5 to 2068 mg/m^3^-years, which was higher in people working in GRG compared with tyres production (maximum LCE 1661 mg/m^3^-years) and was highest for workers in Crude Materials/Mixing compared with those in other departments. LCE to rubber fumes ranged from 1 to 796 mg/m^3^-years, comparable in the GRG (0.6–796.3 mg/m^3^-years) and tyres sectors (0.6–698.0 mg/m^3^-years) and was highest for those workers in the Crude Materials/Mixing (7.2–796.3 mg/m^3^-years).

NDMA-LCE was higher than NMor-LCE and ranged from 1 to 949 µg/m^3^ compared with 0.3 to 125.3 µg/m^3^, respectively, but was comparable for GRG and tyres production. Whereas NDMA LCE was highest for workers in the curing departments, LCE to NMor was highest in workers in the preprocessing and assembly departments.

## Discussion

This study aimed to create a historical quantitative JEM for exposure to rubber dust, rubber fumes and n-Nitrosamines in the UK rubber manufacturing industry for the period 1915–2002 from available quantitative measurements collected between 1977 and 2002 and use this to estimate LCEs for a cohort of rubber workers with 49 years of follow-up (1967–2015). Overall, the results with respect to average exposure levels were broadly in agreement with previous findings from the EXASRUB database,^3 4 9^ but average levels of rubber dust were lower than those reported in Poland.^15^ Other exposure studies for the UK rubber industry examined exposure levels to rubber dust and rubber fumes, but not n-Nitrosamines^2–4^ or compared n-Nitrosamines between different countries alone.^9^ Estimates of average exposures of individual nitrosamines were comparable to those observed previously and showed little variation of individual nitrosamines across departments, which could be attributed to high numbers of measurements below the LOD.^5 9^

Longitudinal trends for rubber dust ranged from −1% per year in Curing to −6.3% per year in Crude Materials/Mixing. More modest declines of rubber dust exposure (−3.9 %/year in crude materials and mixing) were previously found.^4^ Nonetheless, these were comparable to previous trends based on the same data and reported −4.1 %/year for BRMA measurements and −2.3 %/year for HSE-NEDB data in the industry as a whole.^3^ This was also comparable to the average trend in the Dutch industry (−5.7 %/year)^16^ but was about half that reported in the Polish industry, which ranged from-2.5%/year in pre-treating to −12.4 %/year during handling of crude materials.^15^

Temporal trend in rubber fumes ranged from +0.5 %/year to −6.5 %/year in different department groups, which was roughly comparable to the overall −2.9% and −4.8% per year, depending on the data source, has been reported previously.^3^ Differences are likely the result of varied modelling strategies, including larger grouping of departments in our study, which used fewer groups aggregated from different departments, and the fact we used separate models for these department groups instead of estimating from one shared model.

n-Nitrosamines in the European rubber industry were primarily collected in Germany, and these were included in the EXASRUB database^9^ and in these analyses, with some additional cross-sectional data from other countries, including the UK, for which confidence intervals overlapped with German data. As such, it is no surprise that similar trends were observed in our study compared with those reported previously.^9^

Slight increases in annual exposures to rubber fumes, NSS and NDMA (0.5%, 1.9% and 1%, respectively) were observed in the finishing department. However, these increases are not statistically significant (online supplementary table S6) and thus also consistent with no change or reductions. These trends are possibly due to a very low level of exposures with little opportunity for further reductions. Furthermore, exposure sources may not be task-related but rather are due to off-gassing from the cured products. Steep declines in the levels of exposure to n-Nitrosamines within the curing department in 1970–1975 were expected as improvements to the production process and rubber mixtures were made within this period,^17^ particularly to the curing department which is the main source of exposures to nitrosamines.^4^

LCEs have been calculated from estimates of average exposures for each department group and year for which exposure estimates were required. A JEM approach has been used before in epidemiological work in the UK rubber industry, but this was based on expert assessment classifying exposure as low, medium or high,^18^ rather than basing estimates of average exposure on quantitative exposure measurements collected in the industry, as we did in this study. A comparable approach to the one in this study, however, was used to assess the exposure-response association between carcinogenic compounds and cancer mortality for a cohort of Polish rubber factory workers.^15 19^

There are several limitations to this analysis. First, measurements were not uniformly collected and were a compilation of data collected for compliance testing, research or due to worker complaints. We adjusted for personal/stationary measurement type and for source of the data, similar to previous work, but nonetheless this may still have resulted in residual errors in estimates of central tendency because of exposure affecting factors not taken into account. Unequal numbers of measurements from each factory over time may also have affected estimates of the time trends because factories contributed different numbers of measurements each year. However, this will have been to some extent negated by the use of mixed-effects models which accounted for clustering of measurements in factories. Although previous work on grouping strategies for exposure assessment in the rubber industry indicated grouping based on department would be preferable over exposure-determinant based assessment,^20^ this would have resulted in measurement error from not taking into account impact of control measures, such as installation of local exhaust ventilation or other group-level or individual-level determinants that could have affected exposures.^3 20^ We imputed measurements<LOD with a single value assuming a uniform distribution. Simulation studies indicate that this may bias estimates of variability,^21^ but since we were only interested in an estimate of central tendency for the JEM, the impact on LCE is expected to be minimal.

To develop a longitudinal JEM, n-Nitrosamines measurements from Germany were used to supplement the low number of measurements from the UK, which was based on previous comparisons that indicated no evidence of significant differences between both countries.^9^ Comparison of the data that we do have for the same years from both countries was used to provide an adjustment factor, but it is unknown whether this factor would have changed over time.

Models were extrapolated backwards from the first available data in the late 1970s to early 1980s using linear extrapolation of time trends (although capped to avoid unrealistic estimates). Due to lack of quantitative information about exposures in earlier years (1915–1970s), we do not know whether these assumptions are broadly correct, but sensitivity analyses comparing LCE quartiles capped and uncapped show the impact on LCE for epidemiological analyses to be minimal (data not shown). This is because workers who are in the highest quartiles while capped are also in the highest quartiles uncapped and the proportion of data from the highest quartile is 15.7%. Additionally, the proportion of person years contributing to the LCEs from before 1960s was below 10% (data not shown).

The current study used the largest database of exposure measurements for the rubber industry in the UK spanning 25 years, which enabled estimation of quantitative levels and time trends for each exposure. We have also conducted a sensitivity analysis that showed that LCE estimates were quite stable for our British rubber manufacturing workers cohort. Despite the various sources of error described above, the use of group-based estimates of exposure for workers will have likely resulted in epidemiological findings containing Berkson-type error rather than classical errors, resulting in reduced statistical power rather than significant bias in the results.^22^

## Conclusion

This study aimed at developing quantitative job-exposure matrices for exposure to rubber dust, rubber fumes and n-Nitrosamines, to enable estimation of cumulative exposures for a cohort of UK rubber manufacturing workers with 49 years of follow-up for mortality. These analyses indicated differences between exposure levels among departments and demonstrated declines in average exposure across the 1915–2002 time period, the extent of which differed between departments. Overall, the results were broadly in agreement with previous findings from the EXASRUB database^3 4 9^ and from others in the European industry.^2 5^ The observed differentials in levels of cumulative exposure to rubber dust, rubber fumes and n-Nitrosamines could potentially place workers at increased risk for cancer mortality, and this has been investigated in our accompanying papers.^7 14^

**Table 3:** Job exposure matrix for n-Nitrosamines sum score (μg/m^3^)

Year	Crude materials and mixing		Preprocessing and assembly		Curing/Vulcanising		Finishing, assembly and miscellaneous	
	Tyres	GRG	Tyres	GRG	Tyres	GRG	Tyres	GRG
1915	1.91	2.10	394.62	751.30	1291.00	1291.00	0.20	0.19
1920	1.80	1.97	283.15	532.20	1291.00	1291.00	0.22	0.21
1925	1.71	1.85	203.16	377.00	1291.00	1291.00	0.24	0.24
1930	1.62	1.73	145.77	267.10	1291.00	1291.00	0.26	0.26
1935	1.53	1.62	104.59	189.20	1291.00	1291.00	0.29	0.29
1940	1.45	1.52	75.05	134.10	1291.00	1291.00	0.32	0.31
1945	1.37	1.43	53.85	95.00	1291.00	1291.00	0.35	0.35
1950	1.30	1.34	38.64	67.30	1291.00	1291.00	0.39	0.38
1955	1.23	1.25	27.72	47.70	1291.00	1291.00	0.43	0.42
1960	1.16	1.17	19.89	33.80	1291.00	1291.00	0.47	0.46
1965	1.10	1.10	14.27	23.90	1291.00	1291.00	0.52	0.51
1970	1.04	1.03	10.24	16.90	564.80	1097.10	0.57	0.56
1975	0.99	0.97	7.35	12.00	211.30	410.40	0.63	0.62
1980	0.93	0.91	5.27	8.51	79.00	153.50	0.69	0.68
1985	0.88	0.85	3.78	6.03	29.60	57.40	0.76	0.75
1990	0.84	0.80	2.71	4.27	11.10	21.50	0.84	0.82
1995	0.79	0.75	1.95	3.02	4.14	8.04	0.92	0.91
2000	0.75	0.70	1.40	2.14	1.55	3.01	1.01	1.00
2002	0.73	0.68	1.22	1.87	1.05	2.03	1.05	1.04

GRG, general rubber good.

**Table 4:** Job exposure matrix for NDMA and NMor (μg/m^3^)

Year	Crude materials and mixing				Preprocessing and assembly				Curing/Vulcanising				Finishing, assembly and miscellaneous			
	NDMA		Nmor		NDMA		Nmor		NDMA		Nmor		NDMA		Nmor	
	Tyres	GRG	Tyres	GRG	Tyres	GRG	Tyres	GRG	Tyres	GRG	Tyres	GRG	Tyres	GRG	Tyres	GRG
1915	0.17	0.33	0.28	0.12	0.06	0.07	5.83	5.15	71.42	51.47	0.35	0.43	0.16	0.13	2.02	1.65
1920	0.16	0.31	0.27	0.11	0.06	0.08	4.68	4.14	51.19	36.89	0.36	0.43	0.16	0.13	1.73	1.42
1925	0.16	0.30	0.26	0.11	0.07	0.09	3.76	3.32	36.69	26.44	0.36	0.44	0.17	0.14	1.48	1.21
1930	0.15	0.29	0.25	0.10	0.08	0.10	3.02	2.67	26.30	18.95	0.37	0.45	0.18	0.15	1.27	1.04
1935	0.14	0.28	0.24	0.10	0.09	0.11	2.42	2.14	18.85	13.58	0.38	0.46	0.19	0.16	1.09	0.89
1940	0.14	0.27	0.23	0.10	0.10	0.13	1.95	1.72	13.51	9.74	0.38	0.47	0.20	0.16	0.93	0.76
1945	0.13	0.25	0.22	0.09	0.12	0.14	1.56	1.38	9.68	6.98	0.39	0.48	0.21	0.17	0.80	0.65
1950	0.13	0.24	0.21	0.09	0.13	0.16	1.25	1.11	6.94	5.00	0.40	0.48	0.22	0.18	0.68	0.56
1955	0.12	0.23	0.20	0.09	0.15	0.18	1.01	0.89	4.98	3.59	0.41	0.49	0.24	0.19	0.58	0.48
1960	0.12	0.22	0.20	0.08	0.17	0.21	0.81	0.72	3.57	2.57	0.41	0.50	0.25	0.20	0.50	0.41
1965	0.11	0.21	0.19	0.08	0.19	0.23	0.65	0.57	2.56	1.84	0.42	0.51	0.26	0.21	0.43	0.35
1970	0.11	0.21	0.18	0.08	0.21	0.26	0.52	0.46	1.83	1.32	0.43	0.52	0.28	0.22	0.37	0.30
1975	0.10	0.20	0.18	0.07	0.24	0.30	0.42	0.37	1.31	0.95	0.44	0.53	0.29	0.24	0.31	0.26
1980	0.10	0.19	0.17	0.07	0.27	0.34	0.34	0.30	0.94	0.68	0.45	0.54	0.31	0.25	0.27	0.22
1985	0.09	0.18	0.16	0.07	0.31	0.38	0.27	0.24	0.68	0.49	0.45	0.55	0.32	0.26	0.23	0.19
1990	0.09	0.17	0.16	0.07	0.35	0.43	0.22	0.19	0.48	0.35	0.46	0.56	0.34	0.28	0.20	0.16
1995	0.09	0.17	0.15	0.06	0.39	0.49	0.17	0.15	0.35	0.25	0.47	0.57	0.36	0.29	0.17	0.14
2000	0.08	0.16	0.15	0.06	0.44	0.55	0.14	0.12	0.25	0.18	0.48	0.58	0.38	0.31	0.14	0.12
2002	0.08	0.16	0.14	0.06	0.47	0.58	0.13	0.11	0.22	0.16	0.48	0.59	0.38	0.31	0.14	0.11

GRG, general rubber good.
