# Supplementary material for: Job-exposure matrix for historical exposures to rubber dust, rubber fumes and n-Nitrosamines in the British rubber industry
Source: Occup Environ Med. 2019 Feb 16;76(4):259–67. doi: 10.1136/oemed-2018-105182 (PMC6581116; doi:10.1136/oemed-2018-105182)
Supplement: Supplementary data [file oemed-2018-105182supp001.pdf]

ONLINE SUPPLEMENTARY MATERIALS

Table S1. Linear mixed effects models on logged concentrations of rubber dust by department

**Crude materials and mixing**	**β**	**S.E.**	**p-value**	**95% CI**	
Year	-0.065	0.007	<0.001	-0.078	-0.051
Sector (GRG=1; Tyre=0)	0.481	0.158	0.002	0.171	0.791
Sample type (Personal=1; Stationary=0)	0.801	0.065	<0.001	0.673	0.929
Dataset (BRMA=1; HSE-NEDB=0)	-0.287	0.510	0.573	-1.286	0.712
Intercept	128.430	14.043	0.000	100.906	155.953

**Pre-processing and assembly**	**β**	**S.E.**	**p-value**	**95% CI**	
Year	-0.012	0.012	0.312	-0.034	0.011
Sector (GRG=1; Tyre=0)	0.434	0.178	0.015	0.085	0.784
Sample type (Personal=1; Stationary=0)	0.657	0.116	<0.001	0.431	0.884
Dataset (BRMA=1; HSE-NEDB=0)	-0.165	0.501	0.742	-1.148	0.817
Intercept	22.025	23.025	0.339	-23.102	67.153

**Curing/Vulcanizing**	**β**	**S.E.**	**p-value**	**95% CI**	
Year	-0.010	0.009	0.260	-0.028	0.008
Sector (GRG=1; Tyre=0)	0.150	0.135	0.267	-0.115	0.414
Sample type (Personal=1; Stationary=0)	0.803	0.087	<0.001	0.633	0.973
Dataset (BRMA=1; HSE-NEDB=0)	-0.584	0.473	0.217	-1.510	0.343
Intercept	19.644	18.189	0.280	-16.006	55.293

**Finishing, assembly, and miscellaneous**	**β**	**S.E.**	**p-value**	**95% CI**	
Year	-0.012	0.014	0.396	-0.040	0.016
Sector (GRG=1; Tyre=0)	-0.529	0.201	0.008	-0.922	-0.136
Sample type (Personal=1; Stationary=0)	0.998	0.119	<0.001	0.763	1.232
Dataset (BRMA=1; HSE-NEDB=0)	-1.302	0.605	0.031	-2.489	-0.116
Intercept	24.432	28.597	0.393	-31.616	80.481

Table S2. Linear mixed effects models on logged concentrations of rubber fumes by department

Crude materials and mixing	β	S.E.	p-value	95% CI	
Year	-0.067	0.010	<0.001	-0.087	-0.047
Sector (GRG=1; Tyre=0)	0.004	0.118	0.976	-0.228	0.236
Sample type (Personal=1; Stationary=0)	0.481	0.074	<0.001	0.336	0.625
Dataset (BRMA=1; HSE-NEDB=0)	-0.751	0.224	0.001	-1.191	-0.311
Intercept	132.610	20.522	<0.001	92.387	172.833

Pre-processing and assembly	β	S.E.	p-value	95% CI	
Year	-0.025	0.013	0.061	-0.050	0.001
Sector (GRG=1; Tyre=0)	-0.149	0.127	0.240	-0.398	0.100
Sample type (Personal=1; Stationary=0)	0.272	0.099	0.006	0.078	0.465
Dataset (BRMA=1; HSE-NEDB=0)	-0.838	0.523	0.109	-1.863	0.187
Intercept	47.733	26.135	0.068	-3.490	98.957

Curing/Vulcanizing	β	S.E.	p-value	95% CI	
Year	-0.024	0.007	0.001	-0.038	-0.009
Sector (GRG=1; Tyre=0)	0.335	0.086	<0.001	0.167	0.503
Sample type (Personal=1; Stationary=0)	0.680	0.060	<0.001	0.562	0.798
Dataset (BRMA=1; HSE-NEDB=0)	-0.620	0.349	0.075	-1.304	0.063
Intercept	45.770	14.762	0.002	16.836	74.703

Finishing, assembly, and miscellaneous	β	S.E.	p-value	95% CI	
Year	0.005	0.014	0.714	-0.022	0.032
Sector (GRG=1; Tyre=0)	0.148	0.122	0.225	-0.091	0.388
Sample type (Personal=1; Stationary=0)	0.594	0.105	<0.001	0.388	0.801
Dataset (BRMA=1; HSE-NEDB=0)	-1.391	0.515	0.007	-2.400	-0.381
Intercept	-10.946	27.232	0.688	-64.320	42.429

Table S3. Linear mixed effects models on logged concentrations of n-Nitrosamines Sum Score by department

**Crude materials and mixing**	**β**	**S.E.**	**P-value**	**95% CI**	
Year	-0.013	0.013	0.302	-0.038	0.012
Sector (GRG=1; Tyre=0)	-0.070	0.220	0.752	-0.502	0.362
Sample type (Personal=1; Stationary=0)	0.129	0.133	0.333	-0.132	0.390
Country (UK=1; Germany=0)	-0.211	0.200	0.291	-0.603	0.181
Intercept	25.705	24.973	0.303	-23.240	74.651

**Pre-processing and Assembly**	**β**	**S.E.**	**P-value**	**95% CI**	
Year	-0.069	0.021	0.001	-0.111	-0.027
Sector (GRG=1; Tyre=0)	0.301	0.210	0.151	-0.110	0.713
Sample type (Personal=1; Stationary=0)	-0.069	0.179	0.700	-0.419	0.281
Country (UK=1; Germany=0)	1.037	0.325	0.001	0.400	1.674
Intercept	137.269	42.309	0.001	54.346	220.192

**Curing/Vulcanizing**	**β**	**S.E.**	**P-value**	**95% CI**	
Year	-0.197	0.017	<0.001	-0.230	-0.164
Sector (GRG=1; Tyre=0)	0.664	0.092	<0.001	0.484	0.844
Sample type (Personal=1; Stationary=0)	0.465	0.077	<0.001	0.313	0.617
Country (UK=1; Germany=0)	1.699	0.480	<0.001	0.759	2.640
Intercept	390.973	33.437	<0.001	325.439	456.508

**Finishing, Assembly, Miscellaneous**	**β**	**S.E.**	**P-value**	**95% CI**	
Year	0.019	0.015	0.201	-0.010	0.049
Sector (GRG=1; Tyre=0)	-0.014	0.106	0.897	-0.222	0.194
Sample type (Personal=1; Stationary=0)	-0.594	0.126	<0.001	-0.841	-0.346
Country (UK=1; Germany=0)	-0.164	0.249	0.510	-0.652	0.324
Intercept	-37.820	29.995	0.207	-96.610	20.970

Table S4. Linear mixed effects models on logged concentrations of NDMA by department

**Crude materials and mixing**	**β**	**S.E.**	**P-value**	**95% CI**	
Year	-0.009	0.019	0.655	-0.046	0.029
Sector (GRG=1; Tyre=0)	0.659	0.340	0.052	-0.006	1.325
Sample type (Personal=1; Stationary=0)	-0.030	0.203	0.881	-0.428	0.367
Country (UK=1; Germany=0)	-0.518	0.306	0.091	-1.118	0.083
Intercept	15.218	38.260	0.691	-59.769	90.206

**Pre-processing and Assembly**	**β**	**S.E.**	**P-value**	**95% CI**	
Year	0.025	0.019	0.202	-0.013	0.062
Sector (GRG=1; Tyre=0)	0.219	0.201	0.275	-0.175	0.613
Sample type (Personal=1; Stationary=0)	-0.173	0.164	0.293	-0.495	0.149
Country (UK=1; Germany=0)	1.053	0.402	0.009	0.265	1.840
Intercept	-50.684	38.232	0.185	-125.617	24.249

**Curing/Vulcanizing**	**β**	**S.E.**	**P-value**	**95% CI**	
Year	-0.067	0.013	<0.001	-0.091	-0.042
Sector (GRG=1; Tyre=0)	-0.328	0.068	<0.001	-0.461	-0.194
Sample type (Personal=1; Stationary=0)	-0.274	0.057	<0.001	-0.386	-0.162
Country (UK=1; Germany=0)	1.127	0.409	0.006	0.324	1.929
Intercept	131.139	25.258	<0.001	81.634	180.644

**Finishing, Assembly, Miscellaneous**	**β**	**S.E.**	**P-value**	**95% CI**	
Year	0.010	0.018	0.566	-0.025	0.046
Sector (GRG=1; Tyre=0)	-0.211	0.126	0.094	-0.457	0.036
Sample type (Personal=1; Stationary=0)	-0.259	0.142	0.069	-0.538	0.020
Country (UK=1; Germany=0)	0.799	0.360	0.026	0.094	1.504
Intercept	-22.191	35.989	0.537	-92.728	48.346

Table S4. Linear mixed effects models on logged concentrations of NMor by department

**Crude materials and mixing**	**β**	**S.E.**	**P-value**	**95% CI**	
Year	-0.007	0.025	0.771	-0.057	0.042
Sector (GRG=1; Tyre=0)	-0.872	0.451	0.053	-1.756	0.012
Sample type (Personal=1; Stationary=0)	0.358	0.265	0.177	-0.162	0.878
Country (UK=1; Germany=0)	-0.300	0.436	0.492	-1.154	0.555
Intercept	12.819	50.542	0.800	-86.241	111.880

**Pre-processing and Assembly**	**β**	**S.E.**	**P-value**	**95% CI**	
Year	-0.044	0.018	0.014	-0.079	-0.009
Sector (GRG=1; Tyre=0)	-0.124	0.183	0.500	-0.482	0.235
Sample type (Personal=1; Stationary=0)	0.271	0.150	0.070	-0.023	0.565
Country (UK=1; Germany=0)	0.284	0.281	0.314	-0.268	0.835
Intercept	85.364	35.358	0.016	16.064	154.664

**Curing/Vulcanizing**	**β**	**S.E.**	**P-value**	**95% CI**	
Year	0.004	0.012	0.757	-0.020	0.027
Sector (GRG=1; Tyre=0)	0.196	0.065	0.003	0.068	0.324
Sample type (Personal=1; Stationary=0)	-0.252	0.055	<0.001	-0.359	-0.145
Country (UK=1; Germany=0)	1.284	0.358	<0.001	0.582	1.985
Intercept	-9.208	23.954	0.701	-56.157	37.742

**Finishing, Assembly, Miscellaneous**	**β**	**S.E.**	**P-value**	**95% CI**	
Year	-0.031	0.018	0.089	-0.067	0.005
Sector (GRG=1; Tyre=0)	-0.200	0.125	0.109	-0.445	0.045
Sample type (Personal=1; Stationary=0)	-0.204	0.144	0.156	-0.486	0.078
Country (UK=1; Germany=0)	0.109	0.368	0.767	-0.612	0.831
Intercept	60.370	36.356	0.097	-10.886	131.627

Table S5. Number of Rubber dust Measurements by Year and Department

Year	Crude Materials and Mixing	Pre-processing and Assembly	Curing / Vulcanizing	Finishing, Assembly, and Miscellaneous	Total
1977	2	0	1	0	3
1978	6	3	2	3	14
1979	0	0	0	0	0
1980	8	9	8	0	25
1981	14	15	14	2	45
1982	2	5	3	1	11
1983	13	4	7	1	25
1984	23	13	14	17	67
1985	32	0	27	0	59
1986	58	18	40	11	127
1987	106	15	29	12	162
1988	71	12	63	5	151
1989	86	36	40	31	193
1990	52	33	28	53	166
1991	203	90	124	77	494
1992	46	55	50	121	272
1993	91	20	54	18	183
1994	119	23	52	7	201
1995	136	43	94	30	303
1996	252	97	166	70	585
1997	252	42	40	46	380
1998	139	29	17	34	219
1999	86	26	17	25	154
2000	106	13	21	17	157
2001	51	5	12	11	79
2002	51	10	7	14	82
Total	2,005	616	930	606	4,157

Table S5. Number of Rubber fumes Measurements by Year and Department

Year	Crude Materials and Mixing	Pre-processing and Assembly	Curing / Vulcanizing	Finishing, Assembly, and Miscellaneous	Total
1977	2	0	1	0	3
1978	6	3	2	3	14
1979	0	0	0	0	0
1980	2	9	6	0	17
1981	6	7	12	2	27
1982	2	9	3	1	15
1983	4	1	7	1	13
1984	6	10	14	15	45
1985	4	2	46	2	54
1986	17	14	136	9	176
1987	29	27	229	64	349
1988	16	24	158	44	242
1989	42	49	139	40	270
1990	43	39	53	36	171
1991	102	46	133	54	335
1992	28	43	106	21	198
1993	25	18	58	6	107
1994	44	17	88	1	150
1995	79	37	94	16	226
1996	215	82	251	47	595
1997	217	33	103	47	400
1998	68	20	21	25	134
1999	50	14	17	13	94
2000	61	5	21	7	94
2001	29	1	15	7	52
2002	10	5	3	4	22
Total	1,107	515	1,716	465	3,803

Table S5. Number of Nitrosamines Measurements by Year and Department

Year	Crude Materials and Mixing	Pre-processing and Assembly	Curing / Vulcanizing	Finishing, Assembly, and Miscellaneous	Total
1983	9	21	12	19	61
1984	4	0	15	8	27
1985	10	7	13	10	40
1986	0	0	21	14	35
1987	7	27	92	22	148
1988	3	44	266	22	335
1989	8	3	266	9	286
1990	2	3	88	8	101
1991	6	28	214	36	284
1992	3	35	165	45	248
1993	1	13	76	31	121
1994	1	4	0	8	13
1995	2	6	52	21	81
1996	1	9	6	3	19
1997	8	3	54	37	102
1998	9	25	19	13	66
1999	8	19	7	10	44
2000	0	0	0	0	0
2001	3	3	5	1	12
Total	85	250	1,371	317	2,023

Table S6. Annual changes in exposures to rubber dust, rubber fumes, and nitrosamines

Aggregated BRMA Departments	Rubber Dust	Rubber Fumes
Crude materials and Mixing	-6.3%<br>[95% CI: -7.5 to -5.0]	-6.5%<br>[95% CI: -8.4 to -4.6]
Pre-processing and Assembly	-1.2%<br>[95% CI: -3.4 to -1.1]	-2.4%<br>[95% CI: -4.9 to 0.1]
Curing/Vulcanizing	-1.0%<br>[95% CI: -2.8 to 0.8]	-2.3%<br>[95% CI: -3.7 to -0.9]
Finishing, Assembly, and Miscellaneous	-1.2%<br>[95% CI: -4.0 to 1.6]	0.5%<br>[95% CI: -2.2 to 3.2]

Aggregated BRMA Departments	n-Nitrosamines Sum Score	NDMA	NMor
Crude materials and Mixing	-1.3%<br>[95% CI: -3.7 to 1.2]	-0.9%<br>[95% CI: -4.5 to 3.0]	-0.7%<br>[95% CI: -5.6 to 4.3]
Pre-processing and Assembly	-6.7%<br>[95% CI: -10.5 to -2.7]	+2.5%<br>[95% CI: -1.3 to 6.4]	-4.3%<br>[95% CI: -7.3 to -0.9]
Curing/Vulcanizing	-17.9%<br>[95% CI: -20.5 to -15.1]	-6.4%<br>[95% CI: -8.7 to -4.1]	+0.4%<br>[95% CI: -2.0 to 2.8]
Finishing, Assembly, and Miscellaneous	1.9%<br>[95% CI: -1.0 to 5.0]	1.0%<br>[95% CI: -2.5 to 4.7]	-3.1%<br>[95% CI: -6.5 to 0.5]

Table S7. Lifetime Cumulative Exposure (LCE) by Sector and Department (Rubber Dust in mg/m^3/career length)

	N	Mean	Median	S.D.	Min	Max
GRG Sector						
All departments	21,536	69.4	18.7	186.8	1.5	2,068.1
Crude Materials and Mixing	2,588	439.9	313.8	365.7	46.8	2,068.1
Pre-processing and Assembly	3,670	26.4	25.0	11.1	3.3	63.4
Curing/Vulcanizing	3,322	18.4	17.5	7.1	1.5	41.5
Finishing, Assembly, and Miscellaneous	11,956	16.6	15.6	7.4	1.8	40.0
Tyre Sector						
All departments	14,907	48.8	24.9	108.3	2.4	1,660.8
Crude Materials and Mixing	1,164	320.3	245.6	262.1	38.4	1,660.8
Pre-processing and Assembly	4,253	19.2	18.9	6.3	2.6	41.0
Curing/Vulcanizing	1,388	17.1	16.6	5.5	2.4	34.8
Finishing, Assembly, and Miscellaneous	8,102	30.7	29.5	11.5	3.4	67.2
All Sectors						
All departments	36,443	61.0	21.2	159.7	1.5	2,068.1
Crude Materials and Mixing	3,752	402.8	290.8	341.5	38.4	2,068.1
Pre-processing and Assembly	7,923	22.5	21.1	9.6	2.6	63.4
Curing/Vulcanizing	4,710	18.0	17.3	6.7	1.5	41.5
Finishing, Assembly, and Miscellaneous	20,058	22.3	20.0	11.6	1.8	67.2

Table S8. Lifetime Cumulative Exposure (LCE) by Sector and Department (Rubber Fumes in mg/m³/career length)

	N	Mean	Median	S.D.	Min	Max
GRG Sector						
All departments	21,536	17.0	7.2	39.0	0.6	796.3
Crude Materials and Mixing	2,588	77.8	48.5	89.9	7.2	796.3
Pre-processing and Assembly	3,670	7.5	6.5	4.0	0.9	25.1
Curing/Vulcanizing	3,322	20.4	17.8	10.1	1.7	65.0
Finishing, Assembly, and Miscellaneous	11,956	5.7	5.8	2.2	0.6	10.6
Tyre Sector						
All departments	14,907	13.8	6.8	30.8	0.6	698.0
Crude Materials and Mixing	1,164	84.2	62.6	80.6	9.5	698.0
Pre-processing and Assembly	4,253	9.9	9.3	4.2	1.3	29.2
Curing/Vulcanizing	1,388	16.0	14.7	6.8	2.2	44.6
Finishing, Assembly, and Miscellaneous	8,102	5.3	5.3	1.7	0.6	9.0
All Sectors						
All departments	36,443	15.6	7.0	35.9	0.6	796.3
Crude Materials and Mixing	3,752	79.8	51.9	87.1	7.2	796.3
Pre-processing and Assembly	7,923	8.8	8.0	4.3	0.9	29.2
Curing/Vulcanizing	4,710	19.1	16.8	9.5	1.7	65.0
Finishing, Assembly, and Miscellaneous	20,058	5.5	5.6	2.0	0.6	10.6

Table S9. Lifetime Cumulative Exposure (LCE) by Sector and Department (n-Nitrosamines Sum Score in μg/m^3/career length)

	N	Mean	S.D.	Min	Max
GRG Sector					
All departments	21,536	2,826.2	8,296.4	0.5	67,047.5
Crude Materials and Mixing	2,588	16.1	13.1	1.0	71.2
Pre-processing and Assembly	3,670	542.5	833.8	14.8	6586.7
Curing/Vulcanizing	3,322	17689.1	13565.9	1206.5	67047.5
Finishing, Assembly, and Miscellaneous	11,956	5.8	3.7	0.5	15.7
Tyre Sector					
All departments	14,907	1987.6	6894.6	0.5	62934.1
Crude Materials and Mixing	1,164	19.4	13.4	1.1	72.1
Pre-processing and Assembly	4,253	580.2	672.3	13.4	5948.7
Curing/Vulcanizing	1,388	19511.9	13035.2	667.2	62934.1
Finishing, Assembly, and Miscellaneous	8,102	6.9	3.6	0.5	16.2
All Sectors					
All departments	36,443	2483.2	7764.6	0.5	67047.5
Crude Materials and Mixing	3,752	17.1	13.3	1.0	72.1
Pre-processing and Assembly	7,923	562.7	751.6	13.4	6586.7
Curing/Vulcanizing	4,710	18226.3	13436.0	667.2	67047.5
Finishing, Assembly, and Miscellaneous	20,058	6.3	3.7	0.5	16.2

Table S10. Lifetime Cumulative Exposure (LCE) by Sector and Department (NDMA in μg/m³/career length)

	N	Mean	Median	S.D.	Min	Max
GRG Sector						
All departments	21,536	18.2	8.1	44.3	0.8	780.1
Crude Materials and Mixing	2,588	6.7	6.4	2.5	0.9	14.8
Pre-processing and Assembly	3,670	8.7	8.5	3.5	1.0	18.0
Curing/Vulcanizing	3,322	77.5	45.8	92.4	4.5	780.1
Finishing, Assembly, and Miscellaneous	11,956	7.2	7.3	2.7	0.8	13.7
Tyre Sector						
All departments	14,907	19.0	8.8	50.1	0.6	948.8
Crude Materials and Mixing	1,164	3.7	3.6	1.2	0.6	7.6
Pre-processing and Assembly	4,253	7.6	7.4	2.6	1.0	14.1
Curing/Vulcanizing	1,388	12.1	83.4	122.1	12.0	948.8
Finishing, Assembly, and Miscellaneous	8,102	9.4	9.5	3.1	1.0	16.7
All Sectors						
All departments	36,443	18.6	8.4	46.8	0.6	948.8
Crude Materials and Mixing	3,752	5.8	5.3	2.6	0.6	14.8
Pre-processing and Assembly	7,923	8.1	7.8	3.1	1.0	18.0
Curing/Vulcanizing	4,710	90.9	55.1	104.1	4.5	948.8
Finishing, Assembly, and Miscellaneous	20,058	8.1	8.1	3.1	0.8	16.7

Table S11. Lifetime Cumulative Exposure (LCE) by Sector and Department (NMor in µg/m^3/career length)

	N	Mean	Median	S.D.	Min	Max
GRG Sector						
All departments	21,536	12.9	10.6	10.1	0.3	110.7
Crude Materials and Mixing	2,588	2.5	2.4	0.9	0.3	5.4
Pre-processing and Assembly	3,670	20.1	15.0	15.7	2.2	110.7
Curing/Vulcanizing	3,322	17.4	17.5	5.9	1.4	30.4
Finishing, Assembly, and Miscellaneous	11,956	11.6	9.6	7.2	1.2	45.2
Tyre Sector						
All departments	14,907	18.0	15.1	12.1	1.0	125.3
Crude Materials and Mixing	1,164	6.4	6.3	2.1	1.0	12.7
Pre-processing and Assembly	4,253	26.1	22.2	16.1	3.0	125.3
Curing/Vulcanizing	1,388	15.1	15.3	4.3	2.1	25.2
Finishing, Assembly, and Miscellaneous	8,102	15.9	14.1	8.3	1.6	54.9
All Sectors						
All departments	36,443	15.0	12.4	11.2	0.3	125.3
Crude Materials and Mixing	3,752	3.7	2.9	2.3	0.3	12.7
Pre-processing and Assembly	7,923	23.3	19.0	16.2	2.2	125.3
Curing/Vulcanizing	4,710	16.8	16.7	5.5	1.4	30.4
Finishing, Assembly, and Miscellaneous	20,058	13.3	11.3	7.9	1.2	54.9
